# Natural convection in a porous cavity filled (35%MWCNT-65% Fe_3_O_4_)/water hybrid nanofluid with a solid wavy wall via Galerkin finite-element process

**DOI:** 10.1038/s41598-022-22782-0

**Published:** 2022-10-22

**Authors:** Fatima Jasim Gumir, Khaled Al-Farhany, Wasim Jamshed, El Sayed M. Tag El Din, Assmaa Abd-Elmonem

**Affiliations:** 1 Department of Mechanical Engineering, University of Al-Qadisiyah, Al-Qadisiyah, 58001 Iraq; 2 Department of Mathematics, Capital University of Science and Technology (CUST), Islamabad, 44000 Pakistan; 3 Electrical Engineering, Faculty of Engineering and Technology, Future University in Egypt, New Cairo, 11835 Egypt; 4 Department of Mathematics, College of Science, King Khalid University, Abha, Saudi Arabia

**Keywords:** Mathematics and computing, Physics

## Abstract

A numerical analysis of natural convective heat transfer in a square porous cavity with a solid wavy finite wall filled with (35% MWCNT-65% Fe_3_O_4_)/water hybrid nanofluid. The left wavy wall is heated to a constant temperature, the right wall is held at a low temperature, and the top and bottom walls are thermally insulated. Darcy-Brinkman-Forchheimer model is used to model porous medium with hybrid nanofluid. COMSOL Multiphasic Modeling Software via Galerkin finite element method has been used to solve the governing equations. The dimensionless parameters used in this investigation are; modified Rayleigh number (Ra* = 10^2^, 10^3^, 10^4^, and 10^6^), Darcy number (Da = 10^–2^, 10^–4^ and 10^–6^), Solid volume fraction (*ϕ* = 0.01, 0.03, and 0.05),undulation number (*N* = 1, 3, 5, and 7), amplitude of the wavy wall (*A* = 0.1, 0.2, and 0.3), and Prandtl number = 7.2 at constant high porosity. At a high Darcy number (Da = 10^–2^), the isotherm lines parallel to the vertical cavity walls, which means that conduction is the primary method of heat transport. At the same time, the convection mode is increasingly necessary at a lower Darcy number. The convection flow and the maximum amounts of stream function are reduced when both *A* = 0.1 and *N* = 1 increase. The average Nusselt number increases with increasing Ra*, while it decreases with increasing Darcy number and amplitude wave numbers. It has been determined that the largest improvement in heat transfer is at Ra* = 10^4^, Da = 10^–6^, *ϕ* = 0.05, *A* = 0.1, and *N* = 1.

## Introduction

Due to rising energy costs and negative environmental consequences, the need for alternative energy sources is growing. Convection using a hybrid nanofluid is one of the available favorable technologies. The wavy enclosure is used to enhance mass and heat transfer efficiency, as it is used in many applications (e.g., condensers in refrigerators, electric machinery, and solar collectors). In addition, the hybrid nanofluid improved the thermal properties and heat transfer performance, where it has better thermodynamic properties than a single nanofluid. Many studies have involved nanofluids in enhancing heat transfer^1–7^. Hybrid nanofluid is a new type of nanofluid obtained by merging more than one nanofluid. Besides, the hybrid nanofluid improves the thermal properties and heat transfer performance, where these particles, hybrid nanofluid, have better thermodynamic properties than a single nanofluid. Mehryan et al.^8^ studied the natural convection of Al_2_O_3_-Cu/water hybrid nanofluid numerically and experimentally in the porous enclosure. The effect of nanofluid and hybrid-nanofluid on natural/mixed convection with or without a porous media has been presented in many researches^9–20^. Izadi et al.^21^ investigated numerically a natural convection in a ⊥-shaped enclosure filled with (MWCNT-Fe_3_O_4_/water) using the Lattice Boltzmann method. Ruhaniet al.^22^ examined the volume fraction and the temperature effects on the viscosity of the considered (50%ZnO–50%Ag)-Water hybrid nanofluid. A numerical study of entropy generation with (Fe_3_O_4_-CNT)-water hybrid nanofluid in a concentrated horizontal annulus was evaluated by Shahsavaret al.^23^. The temperature of the inner cylinder surface was taken as a constantxtemperature which is more than the outside cylinder's temperature. The Rayleigh number falls between and (10^3^–10^5^), growing a Nu_avg_, friction entropy production rate, and thermal entropy rate by (224.96, 155.25,and 224.65)%, respectively.

Abbasian and Pourmoghadam^24^ experimentally investigated the thermal conductivity behavior of MWCNTS-Al_2_O_3_/ethylene_glycol. The impact of temperature and volume fraction was considered, with temperatures ranging from (25–50 °C) and the volume fraction varied from (0.02–0.8) %. By raising the volume fraction and temperature, it was found that the hybrid nanofluid's thermal conductivity improved. Two correlation equations have been suggested based on experimental data to estimate the hybrid nanofluid's thermal conductivity. The first correlation equation has a maximum inaccuracy of 0.89%, which varies according to the temperature and nanoparticle volume fraction. The second correlation equation consists of 6 models where the temperature was assumed constant, with a maximum error of 0.1–0.8%.

Furthermore, some authors found that using a wavy jar can play an essential role in enhancing heat transfer^25–30^. Al-Kouz et al.^31^ investigated the effects of the wavy wall on natural convection in a tilted cavity filled with water. Uddin et al.^32^ studied a nanofluid's free convective heat transfer in a square container with a wavering upper wall. Abdulkadhim et al.^33^ provide a study of some recent articles in this field of free convection between interior bodies inserted into a variety of complicated cavity forms. The authors concluded that electronic equipment is a practical application for corrugated containers; furthermore, heat transfer is improved by increasing the wave's number and decreasing the amplitude of the waves. A porous media saturated with a hybrid-nanofluid inside a wavy cavity is considered by Dogonchi et al.^34^. Azizul et al.^35^ regarded nanofluid heat transfer inside a square enclosure, including the wavy wall at the top and a heat source below. Local thermal non-equilibrium effects on nanofluid porous medium studied by^36–38^.

The Finite element method (FEM) is a general arithmetical methodology for deciphering PDEs in two or three space variables. This method is very active in all mathematical modeling systems, especially heat transfer and mass transfer. It appeared in Ahmad's^39^ work, where the aim was to pretend the NFs movement and heat arenas inside a motivated geometry occupied by a heat-generating using FEM. Hiba et al.^40^ optimized hybrid NFs utilizing the generalized FEM. Ali et al.^41^ used the approach of FEM to measure the melting effect on CCHFM and heat energy types for allied MHD NFs movement. Abderrahmane et al.^42^ obtained the optimal solution for non-Newtonian NFs employing FEM. Rana and Gupta^43^ prepared a solution for quadratic convective and active movement of hybrid NFs over a revolving pinecone utilizing FEM. Pasha and Domiri-Ganji^44^ analyzed hybrid NFs on widening shallow Chamfer flippers by FEM. Redouane et al.^45^ studied the thermal movement flood of hybrid NFs in animated inclusion with switch cylindrical cavities by assuming generalized FEM. Alrowaili et al.^46^ presented a magnetic radioactive single-minded convection of NFs utilizing FEM. Zaaroura et al.^47^ modeled a dynamic system of NFs by a homogenization technique optimized by FEM. Ahmed and Alhazmi^48^ impacted the revolution and numerous heat conditions of rolls with glass spheres in the company of radioactivity with FEM simulation.

Numerical research into a square enclosure with solid wavy walls containing an (MWCNT-Fe_3_O_4_)/water hybrid nanofluid in a saturated porous media has been conducted. The structure is surrounded by a right wall that is kept at low temperatures, and a heated, wavy wall on the left, while the other walls are adiabatic. The governing equations are Developed and presented in dimensional and dimensionless forms. Governing equations were solved using COMSOL multiphysics modeling software. The parameters that have been used in this study are (Ra* = 10^2^–10^6^), (N = 1, 3, 5, and 7), (A = 0.1, 0.2, and 0.3), (ε = 0.6), (ϕ = 0.01, 0.03, and 0.05), (Da = 10^–2^, and 10^–4^) and (Pr = 7.2). Present data were presented for all instances in the wavy porous cavity, in terms of stream function, isotherms, and average/local Nusselt numbers.

## Mathematical modelling

**Figure 1:**
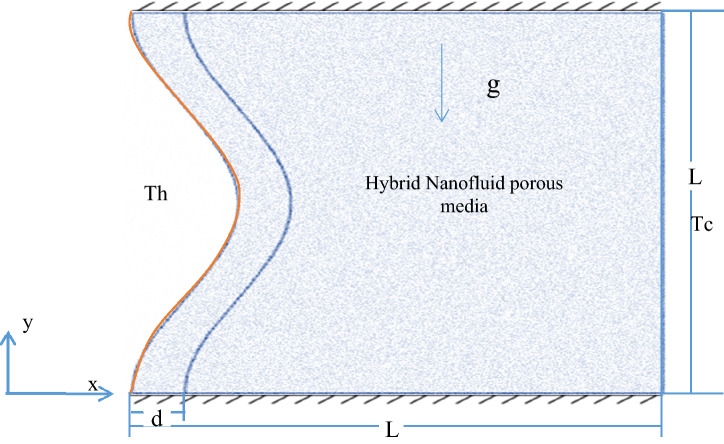
Schematic configuration of the present problem.

Figure 1 illustrates the geometry of the considered problem, which is a 2-D square porous enclosure filled with a hybrid nanofluid with a longitude (L) containing a wavelength on the side of the enclosure. The water is used as the base fluid, and (MWCNT and Fe_3_O_4_) are used as solid nanoparticles.

The wavy left wall remains constant at (T_h_), the right wall is maintained at a low cooling temperature(T_c_), and the horizontal walls are isolated. Boussinesq-approximation was used to model the hybrid nanofluid thermophysical properties. The fluid flow inside the porous medium regime has been simulated using the Darcy–Brinkman–Forchheimer model. The wavy wall has an effective thickness (D = 0.1), and all the enclosure walls are considered solid and impermeable. The hybrid nanofluid with a solid matrix occupies the enclosure's space. The thermophysical characteristics of the base fluid and nanoparticles are listed in Table 1.^49^

**Table 1:** Thermophysical property^49^ of the base fluid and Fe_3_O_4._

Property	K(W/m.k)	ρ(kg/m^3^)	Cp(J/kg.k)
Water	0.613	997	4179
MWCNT	3000	2100	711
Fe_3_O_4_	6	5810	670

The equation of a wavy wall is taken in the following form^26^:

1 $$ X = A\left( {1 - \cos \left( {2N\pi Y} \right)} \right). $$

### Assumption

The following are the assumptions used in the governing equations for natural convection in a porous cavity containing a hybrid nanofluid and solid wavy wall in 2-D Cartesian coordinate:

- Steady-state,
- Incompressible,
- Newtonian fluid,
- All properties remain unchanged except for density, which is adjusted using the Boussinesq approximation,
- Flow is laminar,
- Heat generation is neglected,
- The cavity is considered impermeable.

### The equation of the conservation

Two-dimensional governing equations in Cartesian coordinates for the natural convection of hybrid-nanofluid in a square cavity with a wavy wall are presented in the current work. The dimensional governing equations that were utilized in this study are provided by^50,51^:

2 $$ \frac{\partial u}{{\partial x}} + \frac{\partial v}{{\partial y}} = 0\, $$

3 $$ \frac{1}{\varepsilon }\left( {u\frac{\partial u}{{\partial x}} + v\frac{\partial u}{{\partial y}}} \right) = \frac{\varepsilon }{{\rho_{hnf} }}\left( { - \frac{\partial p}{{\partial x}} + \frac{{\mu_{hnf} }}{\varepsilon }\left( {\frac{{\partial^{2} u}}{{\partial x^{2} }} + \frac{{\partial^{2} u}}{{\partial y^{2} }}} \right) - \frac{{\mu_{hnf} }}{K}u - \frac{{\rho_{hnf} \sqrt {u^{2} + v^{2} } }}{\sqrt K }u} \right) $$

4 $$ \frac{1}{\varepsilon }\left( {u\frac{\partial u}{{\partial x}} + v\frac{\partial u}{{\partial y}}} \right) = \frac{\varepsilon }{{\rho_{hnf} }}\left( \begin{gathered} - \frac{\partial p}{{\partial y}} + \frac{{\mu_{hnf} }}{\varepsilon }\left( {\frac{{\partial^{2} v}}{{\partial x^{2} }} + \frac{{\partial^{2} v}}{{\partial y^{2} }}} \right) - \frac{{\mu_{hnf} }}{K}v - F\frac{{\rho_{hnf} \sqrt {u^{2} + v^{2} } }}{\sqrt K }v \hfill \\ + \left( {\rho \beta } \right)_{hnf} .g\left( {T - T_{c} } \right) \hfill \\ \end{gathered} \right) $$

The energy equation to the fluid region:-

5 $$ u\frac{\partial T}{{\partial x}} + v\frac{\partial T}{{\partial y}} = \frac{{k_{eff} }}{{(\rho Cp)_{hnf} }}\left( {\frac{{\partial^{2} T}}{{\partial x^{2} }} + \frac{{\partial^{2} T}}{{\partial y^{2} }}} \right) $$

The energy equation for the solid wave:-

6 $$ k_{W} \left( {\frac{{\partial^{2} T_{W} }}{{\partial x^{2} }} + \frac{{\partial^{2} T_{W} }}{{\partial y^{2} }}} \right) = 0 $$

The governing equations were transformed using the non-dimensional formula, and the physical characteristics were described by flowing non-dimensional parameters^50,51^:

$$ U = \frac{uL}{\alpha },\;\;V\frac{vL}{\alpha },\;\;X = \frac{x}{L},\;\;Y = \frac{y}{L},\;\;\theta = \frac{{T - T_{{\text{c}}} }}{{T_{h} - T_{{\text{c}}} }} $$

$$ P = \frac{{pL^{2} }}{{\rho_{hnf} \alpha_{f}^{2} }},\;\;Ra = \frac{{g\beta_{f} (T_{h} - T_{c} )L^{3} }}{{v_{f} \alpha_{f} }},\;\;Ra* = Ra \cdot Da,\;\;Pr = \frac{{\nu_{f} }}{{\alpha_{f} }} $$

7 $$ \alpha_{f} = \frac{{k_{f} }}{{\left( {\rho Cp} \right)_{f} }} , Da = \frac{K}{{L^{2} }} , K = \frac{{\varepsilon^{3} d_{p}^{2} }}{{150\left( {1 - \varepsilon } \right)^{2} }} ;\,\,\,F = \frac{1.75}{{\varepsilon^{\frac{3}{2}} \sqrt {150} }} $$

The governing Eqs. (2)–(6) can be written in the following dimensionless format^51,52^:

8 $$ \frac{\partial U}{{\partial X}} + \frac{\partial V}{{\partial Y}} = 0 $$

9 $$ \begin{aligned} \frac{1}{{\varepsilon^{2} }}\left( {U\frac{\partial U}{{\partial X}} + V\frac{\partial U}{{\partial Y}}} \right) & = - \frac{\partial P}{{\partial X}} + \frac{{\mu_{hnf} }}{{\varepsilon \cdot \rho_{hnf} \cdot \alpha_{f} }} \cdot \left( {\frac{{\partial^{2} U}}{{\partial X^{2} }} + \frac{{\partial^{2} U}}{{\partial Y^{2} }}} \right) \\ & \quad - \frac{{\mu_{hnf} }}{{\rho_{hnf} \cdot \alpha_{f} }}\frac{1}{Da}U - \frac{1.75}{{\sqrt {150} }}\frac{{\sqrt {U^{2} + V^{2} } }}{{\sqrt {Da} \cdot \varepsilon^{\frac{3}{2}} }}U \\ \end{aligned} $$

10 $$ \begin{aligned} \frac{1}{{\varepsilon^{2} }}\left( {U\frac{\partial V}{{\partial X}} + V\frac{\partial V}{{\partial Y}}} \right) & = - \frac{\partial P}{{\partial Y}} + \frac{{\mu_{hnf} }}{{\varepsilon \cdot \rho_{hnf} \cdot \alpha_{f} }} \cdot \left( {\frac{{\partial^{2} V}}{{\partial X^{2} }} + \frac{{\partial^{2} V}}{{\partial Y^{2} }}} \right) \\ & \quad - \frac{{\mu_{hnf} }}{{\rho_{hnf} \cdot \alpha_{f} }}\frac{1}{Da}V - \frac{1.75}{{\sqrt {150} }}\frac{{\sqrt {U^{2} + V^{2} } }}{{\sqrt {Da} \cdot \varepsilon^{\frac{3}{2}} }}V + \frac{{(\rho \beta )_{hnf} }}{{\rho_{hnf} \cdot \beta_{f} }}Pr \cdot Ra \cdot \theta \\ \end{aligned} $$

The energy equation to the fluid region:

11 $$ U\frac{\partial \theta }{{\partial X}} + V\frac{\partial \theta }{{\partial Y}} = \frac{{\alpha_{hnf} }}{{\alpha_{f} }}.\frac{{k_{eff} }}{{k_{hnf} }}\left( {\frac{{\partial^{2} \theta }}{{\partial X^{2} }} + \frac{{\partial^{2} \theta }}{{\partial Y^{2} }}} \right) $$

The energy equation for the solid wave:

12 $$ k_{W} \left( {\frac{{\partial^{2} \theta_{W} }}{{\partial X^{2} }} + \frac{{\partial^{2} \theta_{W} }}{{\partial Y^{2} }}} \right) = 0\, $$

### Hybrid nanofluid thermophysical properties

Density (${\rho }_{hnf}$), thermal expansion coefficient(${\alpha }_{hnf}$), heat capacitance (${cp}_{hnf}$), and thermal diffusivity (${\beta }_{hnf}$) are the relationships that define this analysis's hybrid nanofluid effective physical properties. Calculated using the following equations^10,53,54^:

13 $$ \rho_{hnf} = \left( {1 - \phi } \right)\rho_{bf} + \left( {\phi_{MWCNT} .\rho_{MWCNT} + \phi_{Fe3O4} . \rho_{Fe3O4} } \right)\, $$

14 $$ \alpha_{hnf} = \frac{{k_{hnf} }}{{\left( {\rho cp} \right)_{hnf} }} $$

15 $$ \left( {\rho cp} \right)_{hnf} = \left( {1 - \phi } \right)\left( {\rho cp} \right)_{bf} + \left( {\phi_{MWCNT} .\rho_{MWCNT} . cp_{MWCNT} + \phi_{Fe3O4} . \rho_{Fe3O4} . cp_{Fe3O4} } \right) $$

16 $$ \left( {\rho \beta } \right)_{hnf} = \left( {1 - \phi } \right)\left( {\rho \beta } \right)_{bf} + \left( {\phi_{MWCNT} .\rho_{MWCNT} . \beta_{MWCNT} + \phi_{Fe3O4} . \rho_{Fe3O4} . \beta_{Fe3O4} } \right) $$

Experimental data were used to determine dynamic viscosity and thermal conductivity^55^:-

17 $$ \mu_{hnf} = 2.197 - 0.384T^{0.342} + 0.515\phi $$

18 $$ k_{hnf} = 0.464 + 0.024T^{0.537} + 0.442\phi^{0.849} $$

where

19 $$ \phi = \phi_{MWCNT} + \phi_{Fe3O4} $$

Each substance's effective thermal conductivity is dictated by its thermal conductivity and porosity:

20 $$ k_{eff} = \left( {1 - \varepsilon } \right)k_{sp} + \varepsilon k_{hnf} $$

### Nusselt number

The local and average Nusselt number on the wavy wall is written as^56^ to examine the impact of different parameters on heat transport:

21 $$ Nu_{L} = \frac{{k_{eff} }}{{k_{f} }}\frac{\partial \theta }{{\partial Y}} $$

22 $$ \overline{Nu}_{hnf} = \frac{1}{L}\mathop \smallint \limits_{0}^{L} Nu_{L} dL $$

### Boundary conditions

The following shows the non-dimensional boundary conditions.

$$ at\,the\,\,left\,\,wavy wall\,\,for\,\,\,X = \, A(1 \, - \, cos(2N\pi .Y),\,\,\,\,\,\theta = 1 $$

$$ at \, the{\text{ left wavy }}wall \, of\,the \, porous \, cavity,\,\,X = D + \, A(1 \, - \, cos(2N\pi .Y),\,\,\,\frac{{\partial \theta_{w} }}{\partial X} = \frac{{\partial \theta_{{}} }}{\partial X}, \, U = 0, \, and \, V = 0 $$

$$ at\,right\,vertical\,wall\,for\,\,\,X = 1,\,\,\,\,\,\theta = 0, \, U = 0, \, and \, V = 0 $$

23 $$ at\,horizontal \, walls\,\,for\,\,\,Y = 0, \, 1,\,\,\,\,\,\frac{\partial \theta }{{\partial Y}} = 0, \, U = 0, \, and \, V = 0 $$

## Numerical solutions: Galerkin finite element method

The Multiphysics COMSOL (5.6) program is used to resolve the non-dimensional governing Eqs. (8)–(12) using the boundary conditions in Eq. (23). The following physics are chosen in the model component using COMSOL's available physics^56^. Lagrange elements were used to ensure numerical solution stability. Five different meshes have been used to test grid size tolerance to achieve a high-accuracy solution while reducing measurement time with a limited number of components, as seen in Table 2.

**Table 2:** Mesh independence Ra = 10^6^ , Da = 10^–4^ , ϕ = 0.01, ε = 0.6, and pr = 7.2.

Grid size	*Nu*_*avg*_ *at N* = *1*	Grid size	*Nu*_*avg*_ *at N* = *3*	Grid size	*Nu*_*avg*_ *at N* = *5*	Grid size	*Nu*_*avg*_ *at N* = *7*
1097	9.3464	3147	5.4718	7356	2.7935	11,811	1.4183
1630	9.3208	4001	5.5134	9277	2.8226	16,004	1.4401
2579	9.3539	5802	5.5213	13,333	2.8293	22,550	1.447
7196	9.3741	9353	5.5599	18,426	2.8503	30,236	1.4616
19,115	9.3766	21,211	5.5978	34,659	2.8643	55,163	1.4687

**Figure 2:**
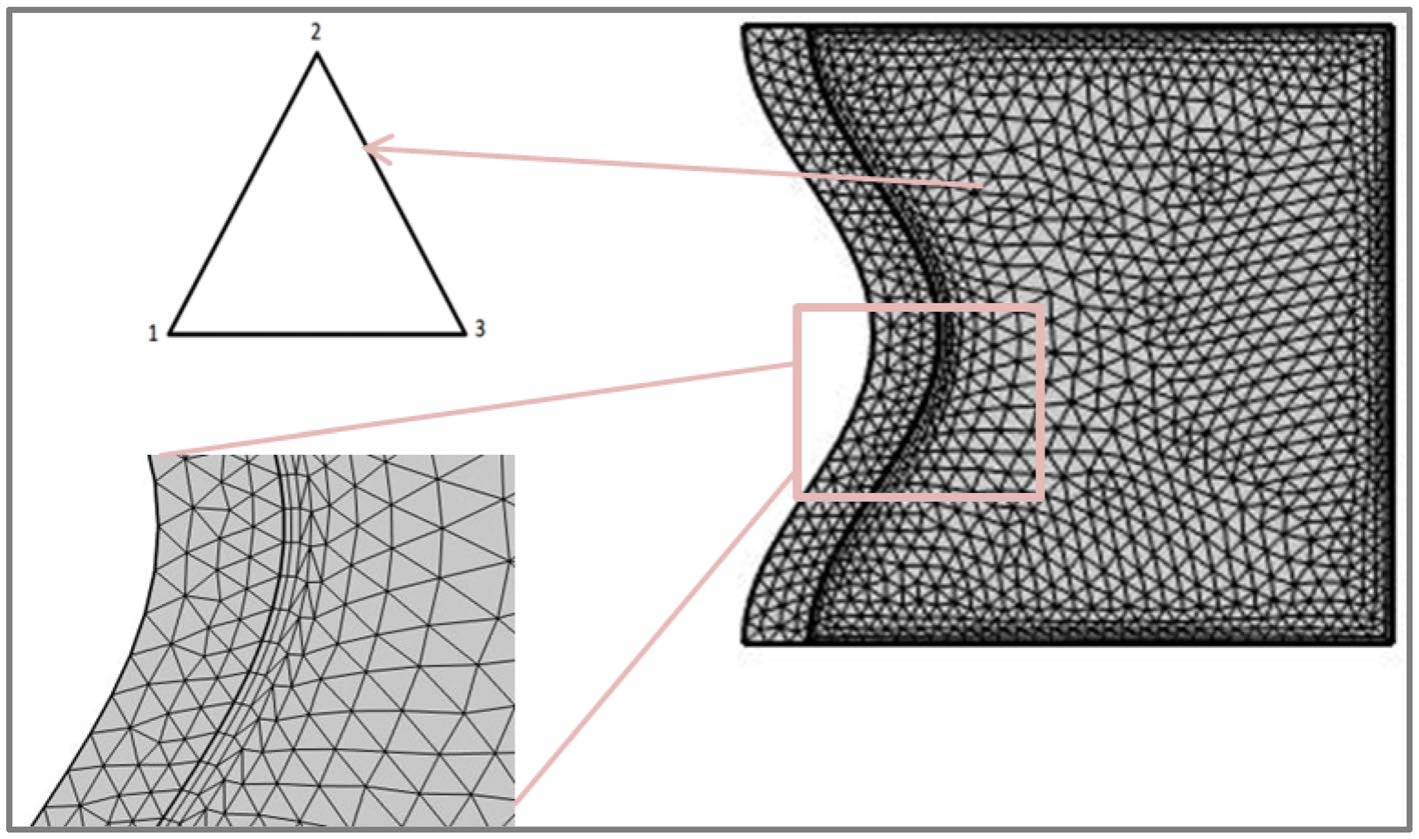
Grid distribution of the computational domain.

Figure 2 depicts a two-dimensional computational domain in a cartesian coordinate system, segmented into many small elements, where the full mesh consists of 2402 domain elements and 177 boundary elements.

### Validation of numerical code

**Figure 3:**
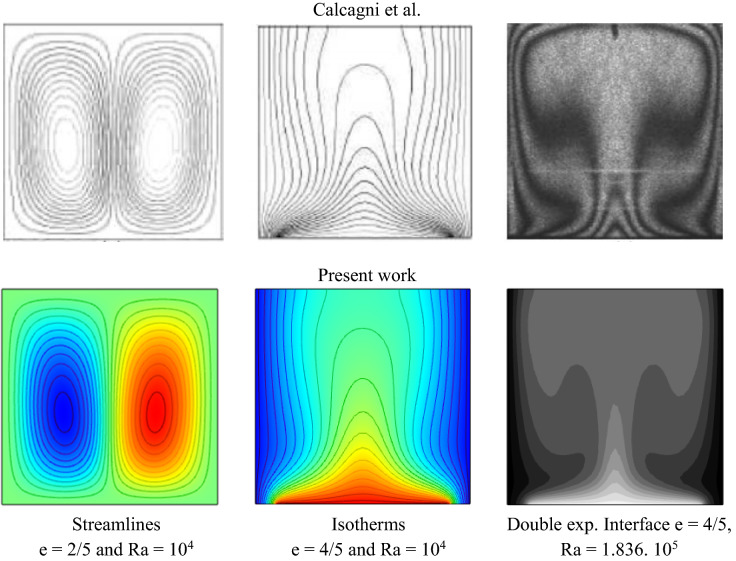
Compared with experimental and numerical results of Calcagni et al.^57^.

Three cases have been used to evaluate the current code accuracy. The comparison of the natural convective heat transfer in a square structure, hot from below and cold from vertical walls, is provided to the numerical and experimental results of Calcagni et al.^57^, as seen in Fig. 3.

**Figure 4:**
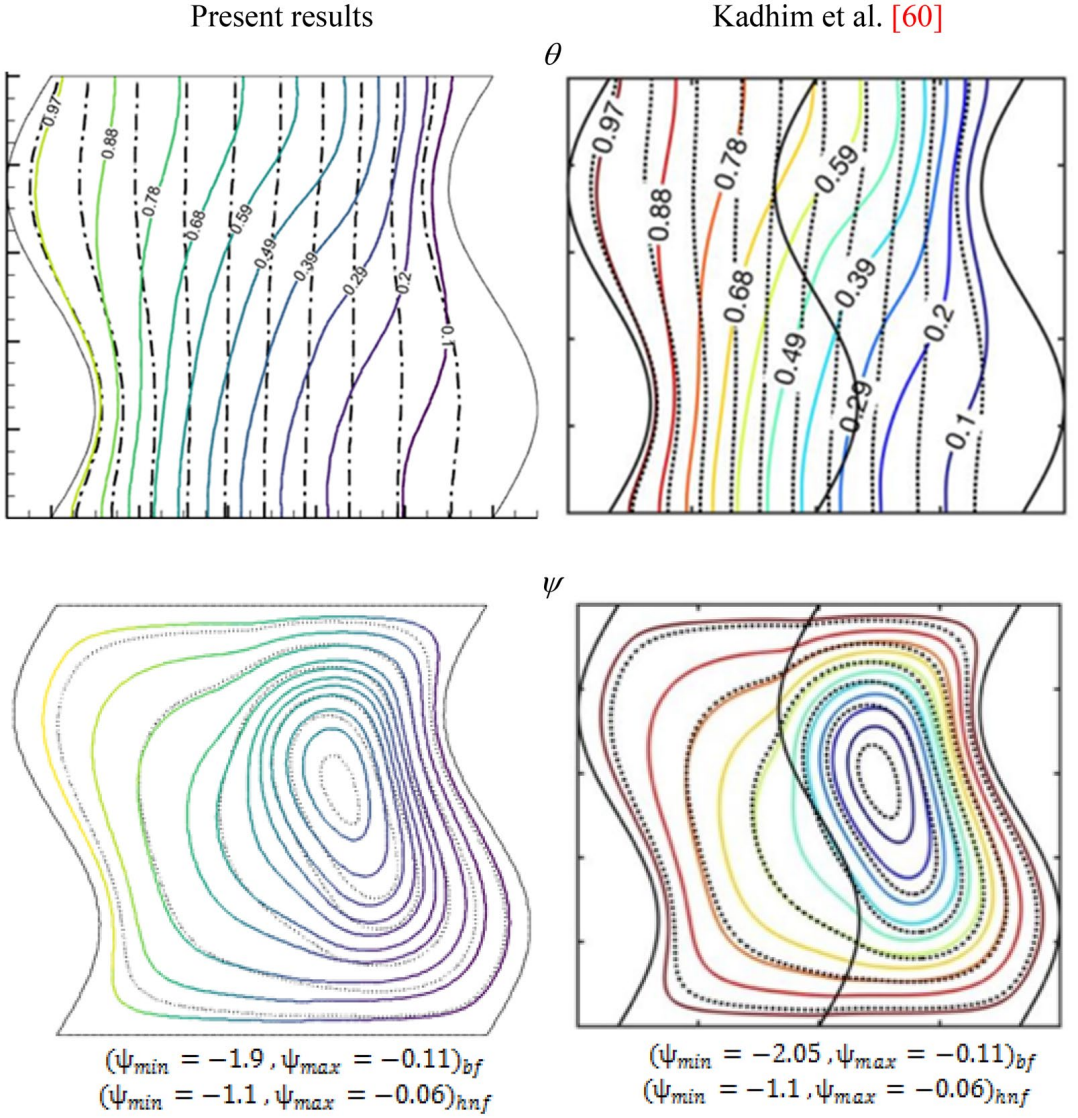
Comparison the streamlines and isotherms of present and Kadhim et al.^58^ study, at Ra = 10^5^, and ϕ = 0.1(solid line) ϕ = 0 (dash line).

Figure 4 and Table 3 provide another comparison to the current code results with the natural convection of Kadhim et al.^58^ using Cu-Al_2_O_3_ hybrid nanofluid within the wavy enclosure. The findings obtained from Kadhim et al.^58^ are considered to support the CFD predictions of the current solver. Figure 4 presented the streamlines and isotherms at Ra = 10^5^ andϕ = 0.1(solid line) ϕ = 0 (dash line), comparisons with the forecasts achieved using the present numerical procedure. Table 3 shows the Nusselt number on the hot wall at Ra = 10^6^,Da = 10^–3^,ϕ = 0.2,N = 1 for the current and Kadhim et al. study. It can be seen there was an intense match with Kadhim et al. study.

**Table 3:** Comparison of the average Nusselt number with Kadhim et al.^58^ for Al_2_O_3_/water nanofluid and Cu-Al_2_O_3_/water hybrid nanofluid at Da = 10^–3^, N = 1.

φ	*Ra*	Average Nusselt number (Nu_avg_)			
		Al_2_ O_3_ /water		Cu-Al_2_ O_3_ /water	
		Kadhim et al.^58^	Present study	Kadhim et al.^58^	Present study
0.1	10^5^	3.343	3.5172	3.430	3.5974
	10^6^	9.083	9.3520	9.302	9.5402
	10^7^	20.026	20.990	20.400	21.332
0.2	10^5^	3.531	3.9089	3.714	4.0942
	10^6^	9.982	10.030	10.489	10.505
	10^7^	23.262	24.048	24.300	24.963

**Figure 5:**
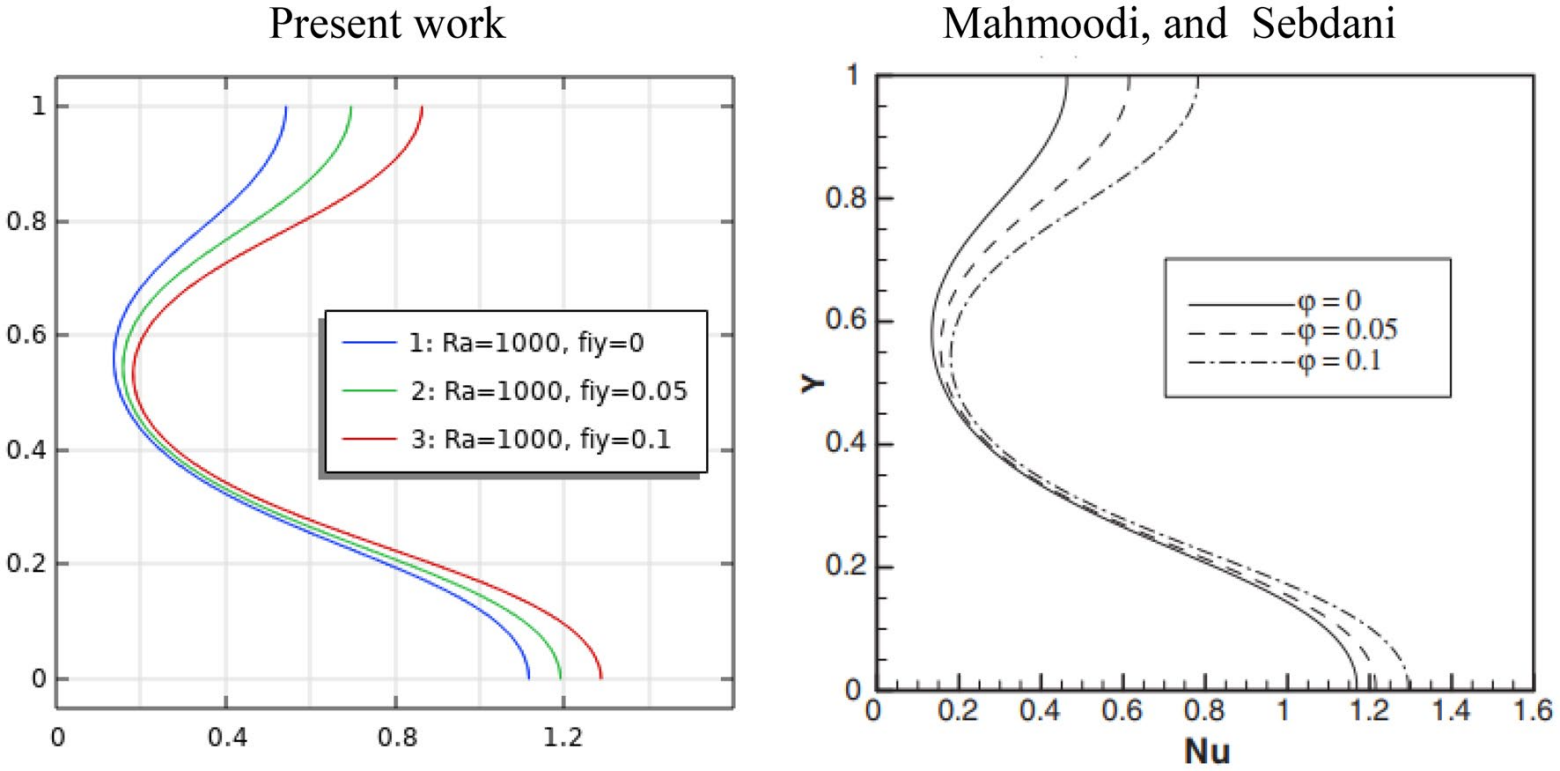
Comparison of the local Nusselt number with Mahmoodi and Sebdani's^59^ study at aspect ratio equal 0.6.

To further raise the confidence in the present numerical findings, another comparison was made with the previous numerical results of Mahmoodi and Sebdani^59^ in the natural convection of Cu-water nanofluid inside a square—cavity with central adiabatic square bodies. Figure 5 illustrates the local Nusselt number with volume fractions of nanoparticles at Ra = 10^3^ and aspect ratio = 0.6, and the present findings are generally well-conformed with those published.

## Result and discussion

Numerical analysis of natural convection heat transfer of hybrid nanofluid within the porous cavity with a solid wavy wall on the left side has been investigated in this study. The left wavy wall is heated, the upper and lower walls are heat-isolated, and the right wall is held at a cold temperature. The study's parameters are Ra, N, A, ε, ϕ, Da, Kr, and Pr. For this analysis the values of the considered parameters are (Ra* = 10^2^, 10^3^, 10^4^, and 10^6^), (N = 1, 3, 5, and 7), (A = 0.1, 0.2, and 0.3), (ε = 0.6), (ϕ = 0.01, 0.03, and 0.05), (Da = 10^–2^, 10^–4^, and 10^–6^), (kr = 1), and (Pr = 7.2).

### Effect Ra* and Darcy number (Da)

**Figure 6:**
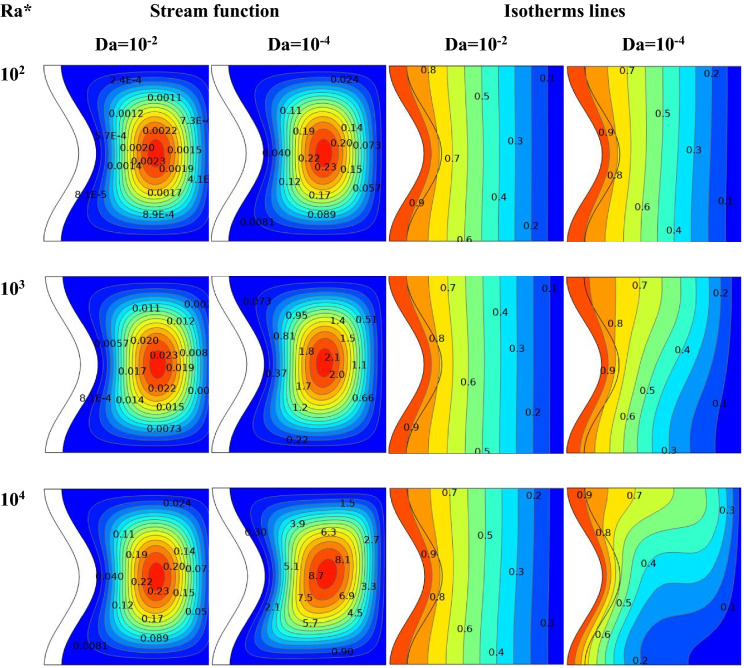
For different values of Da, the streamline and isotherm contours of Ra* are seen (ϕ = 0.01 ε = 0.6 kr = 1 A = D = 0.1 and N = 1).

Figure 6 expresses the impacts of stream function and isotherms line in an enclosure at Ra* of range (10^2^–10^4^) with various Darcy numbers (10^–2^ and 10^–4^), at A = 0.1, N = 1, and **ϕ** = 0.01. The two columns display the stream function on the left and the isotherm on the right.

The isotherms line for all Ra* at Da = 10^–2^ is close to vertical lines. Furthermore, the isotherms at a high amount of Da are parallel to the vertical axis, indicating that conduction is the primary mechanism of heat transport at high Darcy number values. In contrast, by decreasing Da, the heat transfer convection mechanism becomes more important.

When Ra* is increased from 10^2^ to 10^4^ at Da = 10^–4^, the isotherms line changes because the fluid near the wave is heated and flows in the curve form; increasing Ra* decreases the solid wavy wall temperature, indicating improvement in the heat transfer rate from the fluid. Increasing Ra* indicates that the convective flow intensifies with intense isothermal distortions within the fluid. At Da = 10^–2^, when Ra* increases from 10^2^ to 10^4^, the stream function increases from ψ_max_ = 0.0023 to ψ_max_ = 0.23 for a hybrid nanofluid. For Da = 10^–4^, when Ra* increases from 10^2^ to 10^4^, the stream function increases from ψ_max_ = 0. 23 to ψ_max_ = 8.7 for hybrid nanofluid. The Darcy number was observed to impact the stream function significantly. The flow strength and buoyancy force are at a very low Darcy number (Da = 10–4).

**Figure 7:**
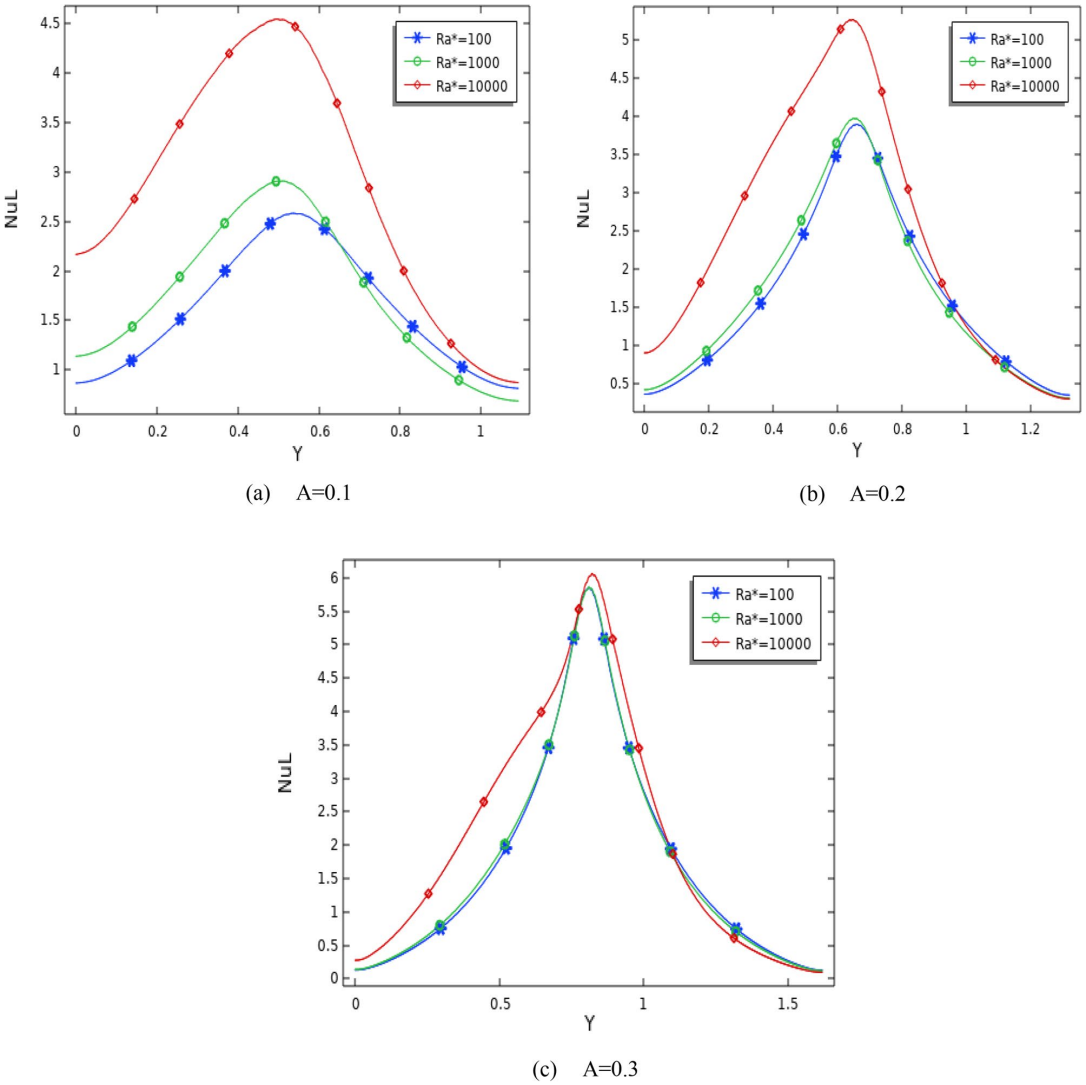
Divergence of Local Nusselt number on the hot wall for various Ra* and Da at number of waves (N = 1), ϕ = 0.01, and (**a**) A = 0.1, (**b**) A = 0.2, and (**c**) A = 0.3

**Figure 8:**
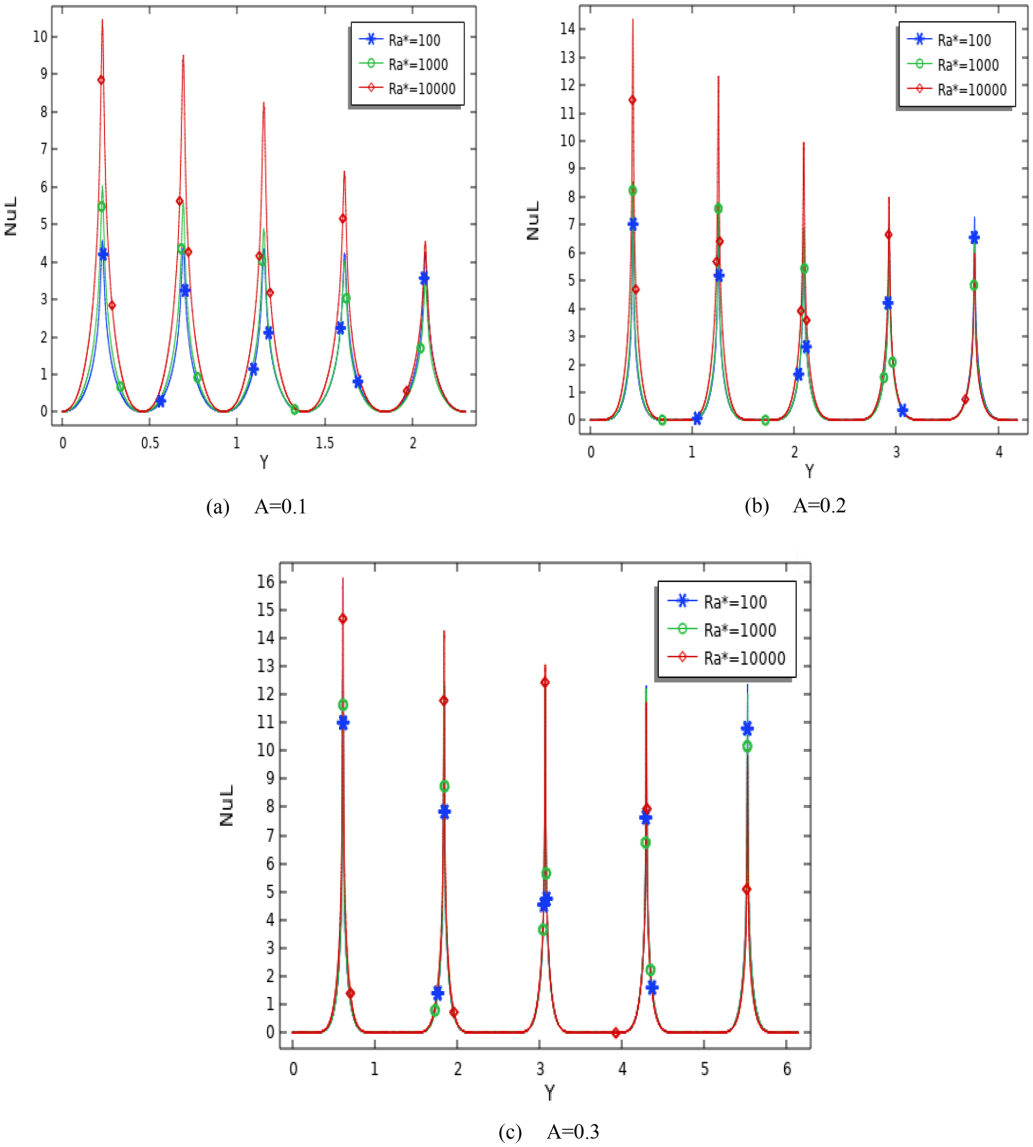
Difference of Local Nusselt number with hot wavy wall for different Ra* and Da at number of waves (N = 5), ϕ = 0.01, and (**a**) A = 0.1, (**b**) A = 0.2, and (**c**) A = 0.3.

The relationship between the local Nusselt number and Ra* on the wavy surface is shown in Figs. 7 and 8 at ϕ = 0.01, Da = 10^–4^, (A = 0.1, 0.2, 0.3), and (N = 1, 5). The heat transfer increases with increasing Ra*, which improves with the amplitude and the number of waves. It is apparent that when the Ra* changes from 10^2^ to 10^4^, the maximum production for Ra = 10^4^.

**Figure 9:**
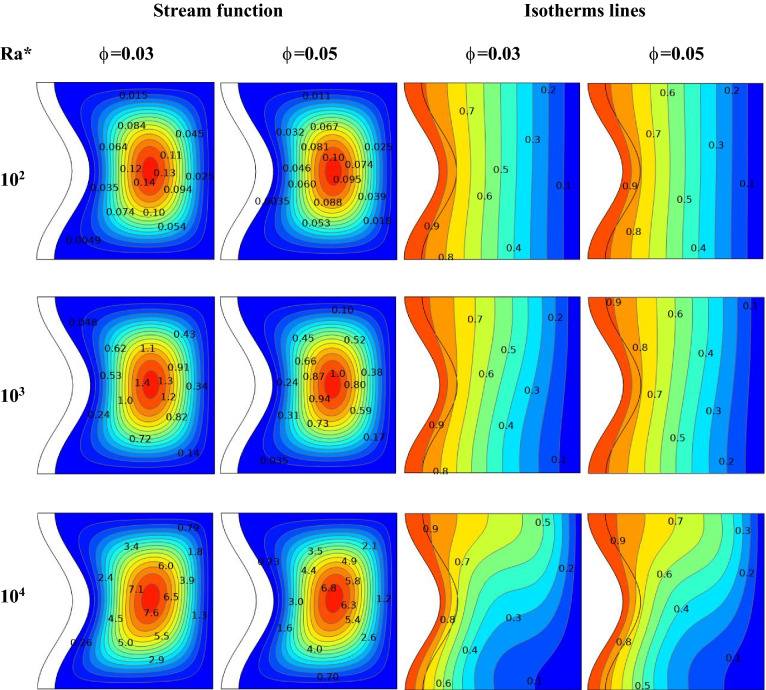
For varying values of solid volume fraction ϕ, the streamline and isotherm contours of Ra* are seen (Da = 10^–4^ ε = 0.6 kr = 1, A = D = 0.1 and N = 1).

### Effect of Ra* and nanoparticles volume fraction

Figure 9 Explains the impact of a set of nanoparticles concentrations(ϕ = 0.03, and 0.05), where Da = 10^–4^, ε = 0.6, A = 0.1, and N = 1on the stream function and isotherm lines of the square enclosure by Ra*. It is found that there is a slight alteration in a stream function and isotherm lines at Ra* = 10^2^ to 10^4^ for all ranges of the nanoparticles volume concentration. Adding nanoparticles increases the viscosity and thermal conductivity of the fluid. It was noted that for ϕ = 0.03, when Ra* increases from 10^2^ to 10^4^, the stream function increases from ψ_max_ = 0.14 to ψ_max_ = 7.6 for hybrid nanofluid, respectively. For ϕ = 0.05, as the Ra* increases from 10^2^ to 10^4^, the stream function increases from ψ_max_ = 0. 1 to ψ_max_ = 6.8 for hybrid nanofluid. It was determined that a rise in Ra* causes an intensification of the convection flow, whereas an increase in ϕ only slightly attenuates the convective flow. In addition, increasing the concentration of nanoparticles leads to an increase in thermal conductivity and dynamic viscosity; thus, it will decrease flow intensity.

### Effect of Ra* and amplitude of a wave

**Figure 10:**
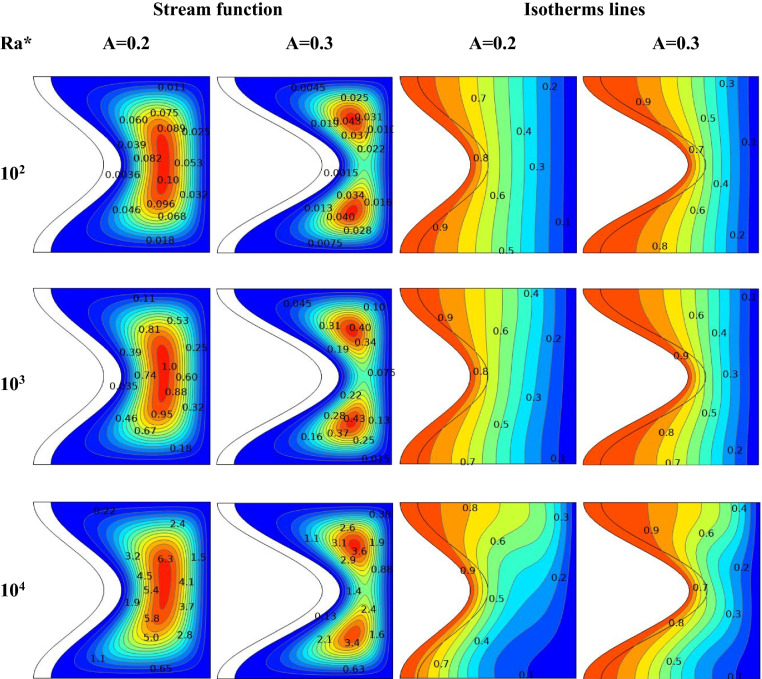
For various values of′ amplitude of wave A, the streamline, and isotherm contours for various Ra* at ϕ = 0.01 ε = 0.6 Da = 10^–4^ kr = 1 D = 0.1 and N = 1.

For saturated porous media/hybrid nanofluid for a range of Ra* at A equal to 0.2 and 0.3, respectively, Fig. 10 illustrates stream function and Isotherm lines at Da = 10^–4^, ϕ = 0.01, ε = 0.6, kr = 1, and N = 1.

Isotherm lines distribution in the interior fluid in both right columns are parallel to the vertical walls, which means most heat is transferred through conduction. The growth of wave amplitude increases the temperature of the solid wave wall, which reduces the heat transfer by the liquid. Also, it can be seen that when A is changed, the fluid flow follows the structure's geometry. In particular, as A increases, the maximum stream function values are decreased, while the minimum values are improved in this Ra* range. For A = 0.2, as the Ra* increases from 10^2^ to 10^4^, the stream function increases from ψ_max_ = 0.1 to ψ_max_ = 6.3 for hybrid nanofluid. For A = 0.3, as the Ra* increases from 10^2^ to 10^4^, the stream function increases from ψ_max_ = 0.043 to ψ_max_ = 3.6 for hybrid nanofluid. Further, increasing the A leads to decreasing the stream function, while increasing the Ra* improves the amount of maximum stream function. The isotherm contours line shifted through rising Ra* from 10^2^ to 10^4^, the hybrid nanofluid in contact with the wave being heated.

### Effect of Ra* and number of waves

**Figure 11:**
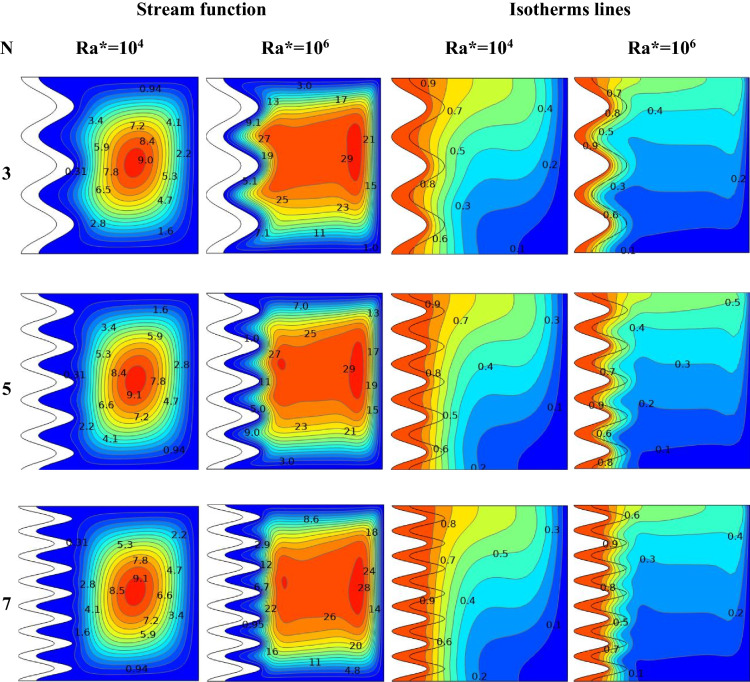
Streamline and isotherm contours for the number of waves N, for numerous Ra* values at ϕ = 0.01 ε = 0.6 Da = 10^–4^ kr = 1 A = D = 0.1.

**Figure 12:**
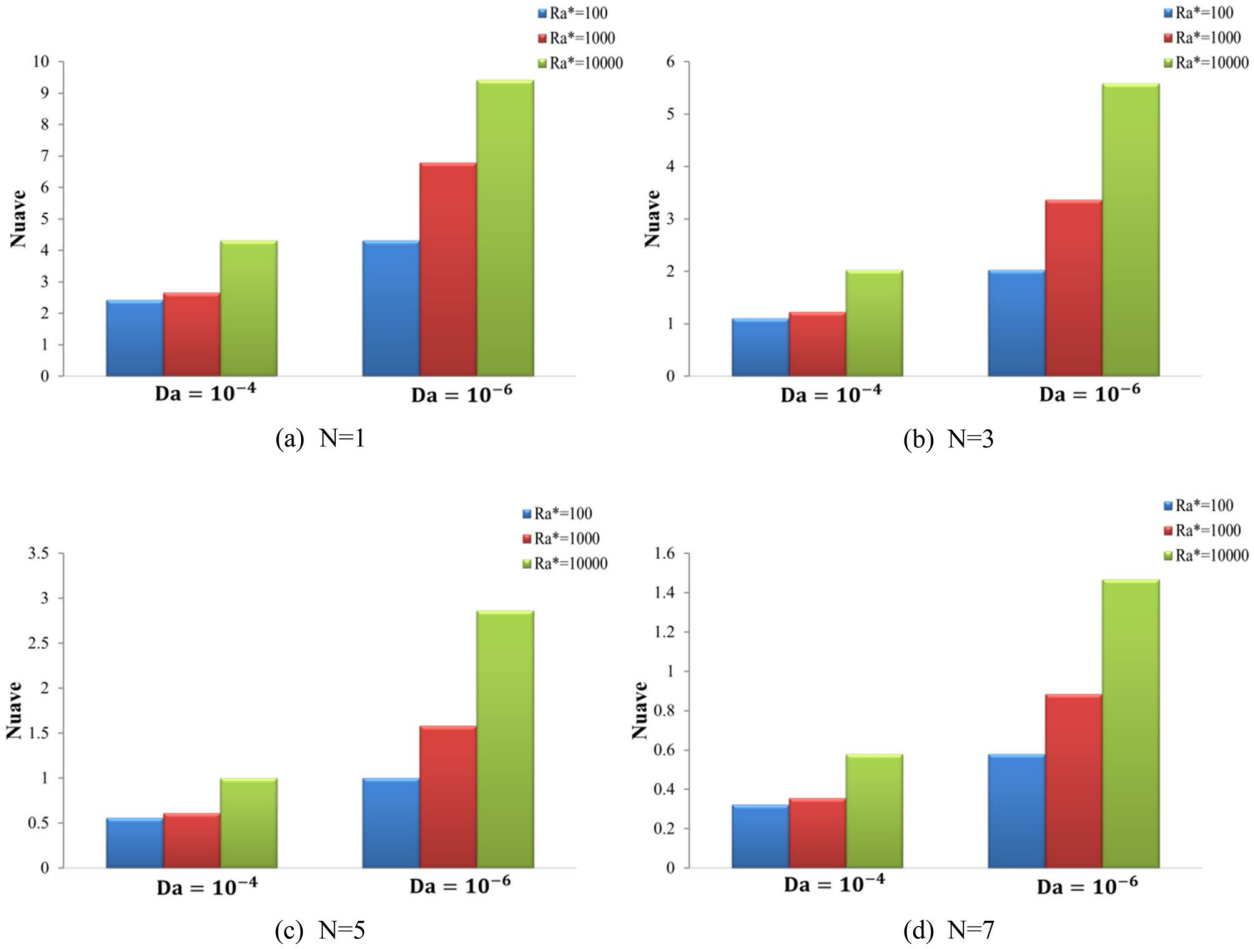
Variation of average Nusselt number (Nu_ave_) with hot wavy wall for diverse Ra* and Da at A = 0.1, ϕ = 0.01, and (**a**) N = 1, (**b**) N = 3, (**c**) N = 5, and (**d**) N = 7.

**Figure 13:**
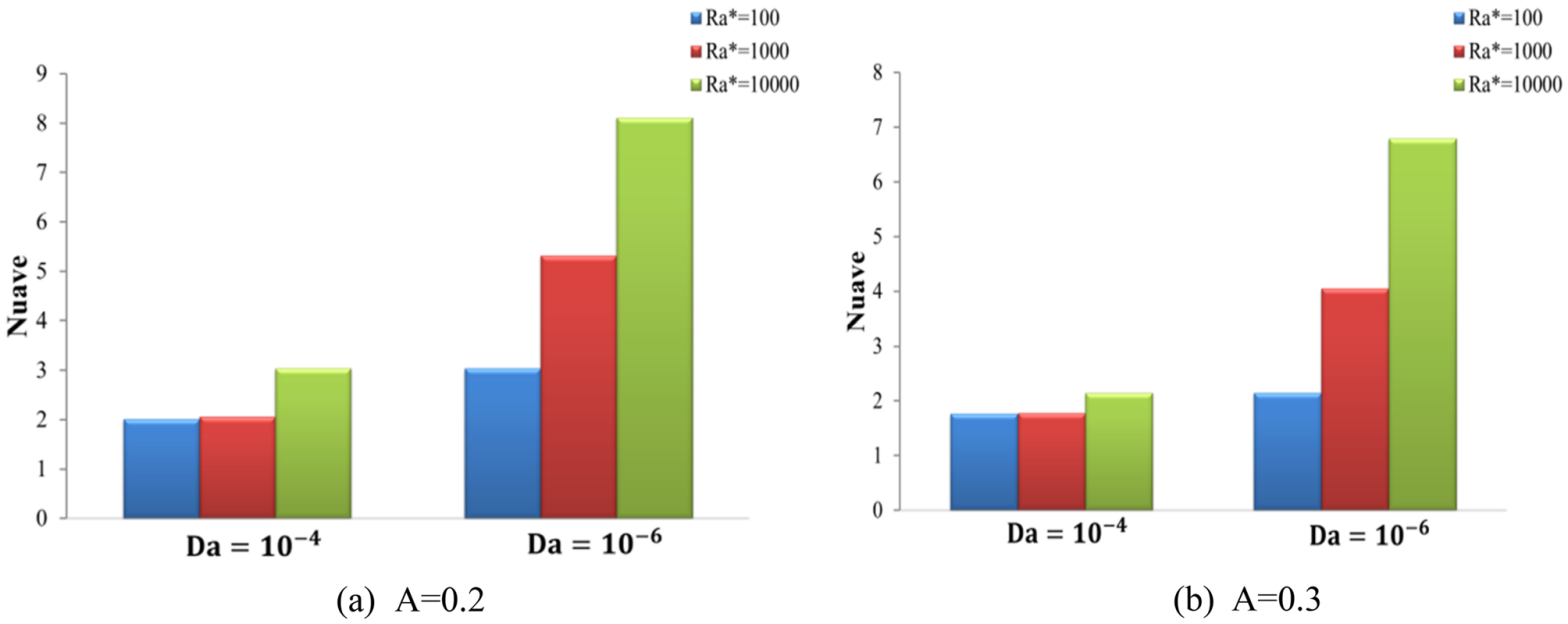
Difference of average Nusselt number (Nu_ave_) with wavy wall for various Ra* and Da at N = 1, ϕ = 0.01, and (**a**) A = 0.2, (**b**) A = 0.3.

**Figure 14:**
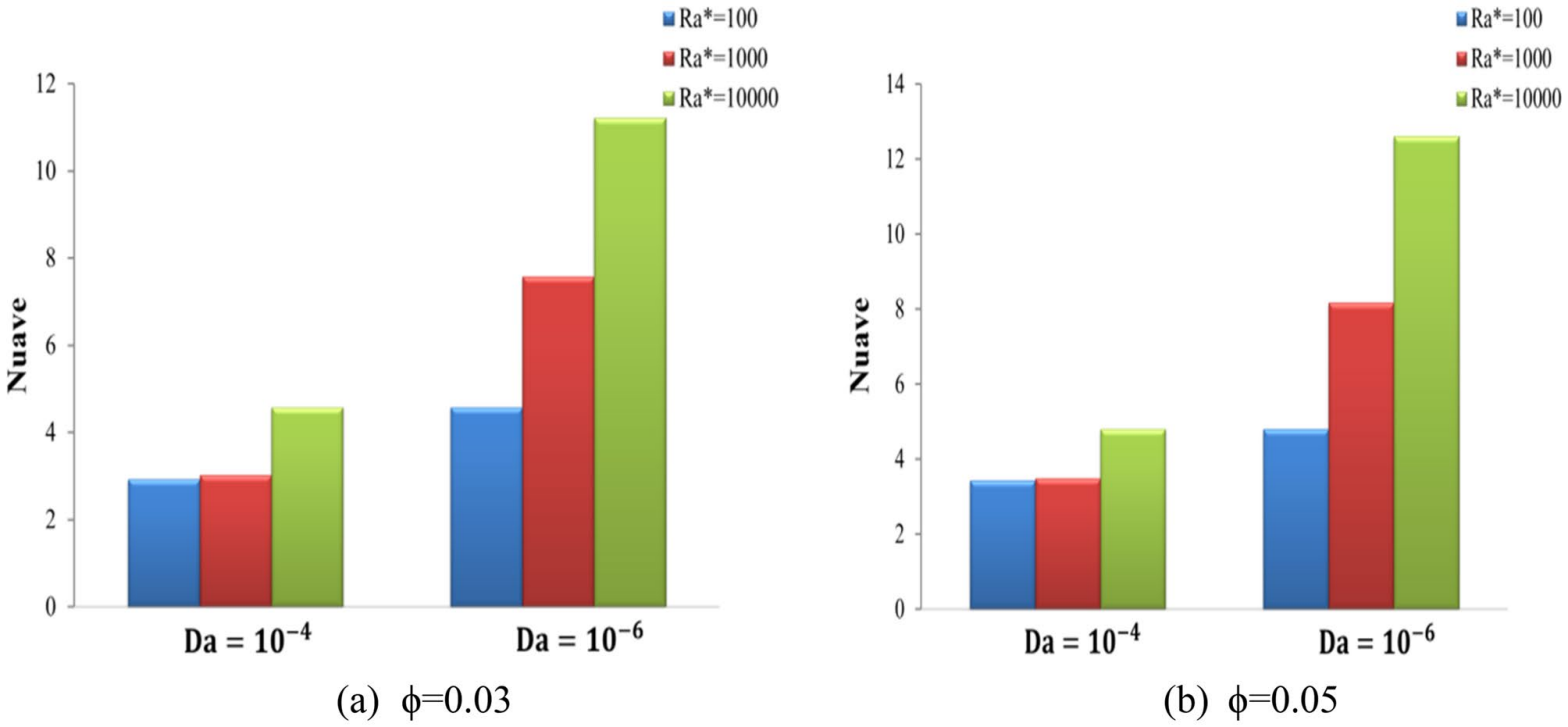
Variation of average Nusselt number (Nu_ave_) on the wavy wall for different Ra* and Da at N = 1, A = 0.1, and (a) ϕ = 0.03, (b) ϕ = 0.05.

Figure 11 displays the Streamlines and isotherms contour lines for N at different Ra* (Ra* = 10^4^ to 10^6^ ) at fixed values of Da = 10^–4^, ϕ = 0.01, and A = 0.1. Various waveforms produce varying stream function patterns and the distribution of temperatures in the cavity. Increasing Ra* contributes thus both to an improvement in buoyancy strength and free convection, thus enhancing stream function. For Ra* = 10^4^, as the N increases from 3 to 7, the stream function increases from ψ_max_ = 9 to ψ_max_ = 9.1 for hybrid nanofluid. For Ra = 10^6^, as the N increases from 3 to 7, the stream function decreases from ψ_max_ = 29 to ψ_max_ = 28 for hybrid nanofluid. Consequently, it was discovered that an increase in Ra* causes the convection flow to intensify, while an increase in (N) causes the convection flow to weaken. It was noticed that growing numbers of waves decrease both heat and fluid flow strength. The effects of Ra* and wave numbers on the $\overline{Nu}_{hnf}$ are shown in Fig. 12**,** at A = 0.1, ϕ = 0.01, and N = (1, 3, 5, and 7). The impact of N also improves by increasing the Ra*. It increases as Ra* increases and reduces with the increase in wave numbers. The heat transfer greatest increase at Ra* = 10^4^ at N = 1. Figure 13 shows the effect of Ra* and wave amplitudes on the average amount of Nusselt in N = 1, ϕ = 0.01, and A = (0.2 and 0.3). Maximum improvement of the average Nusselt number is when Ra* increases from 10^2^ to 10^4^ at Da = 10^–6^ and A = 0.2. The impacts of the nanoparticle volume fractions are seen in Fig. 14, at A = 0.1, N = 1. As ϕ rises, the Nu_ave_ increases slightly with Ra* increasing. The greatest improvement in average Nu at Ra* (10^4^), Da (10^–6^), and ϕ (0.05) is (40.87%) compared to pure fluid.

## Conclusions

In this paper, natural convection was studied numerically utilizing a hybrid (35%MWCNT-65% Fe_3_O_4_)/water nanofluid inside a two-dimensional enclosure with a finite solid wavy wall. The wavy left wall is hot, the right vertical wall is cold, and the horizontal walls are insulated. The governing equations transform into the dimensionless formula and are solved by Multiphysics COMSOL (5.6). The Pr = 7.2, D = 0.1,ε = 0.6,Da = 10^–2^, 10^–4^, and 10^–6^, Ra* = 10^2^, 10^3^, 10^4^, and 10^6^, ϕ = 0.01, 0.03, and 0.05, A = 0.1, 0.2, and 0.3 and a number of waves N = 1, 3, 5, and 7. are investigated.

Drawn-out points:

- The isotherm lines at a higher Darcy value parallel the vertical wall, indicating that conduction is the dominant heat transfer mechanism. In comparison, the convection mode becomes more important at a lower value of Da.
- Increasing the Ra* increases the buoyancy force and the natural convection effect, increasing the stream function.
- The rate of heat transfer increases by rising Ra* and decreasing Da. Furthermore, the results show a slight improvement in the heat transfer rate as Da is reduced from 10^–2^ − 10^–4^. However, the heat transfer rate differs significantly by changing the Da from 10^–4^ to 10^–6^.
- Stream function decreases as the ϕ grows since the addition of nanoparticles increases the fluid's dynamic viscosity. The concentration of nanoparticles affects the average Nusselt number, increasing with concentration.
- Increasing wave amplitude causes a decrease in the maximum stream function values while the minimum values are increased. The temperature of the solid wave wall increases with the wave's amplitude. Consequently, with increased wave amplitude, the $\overline{Nu}_{hnf}$ decreases.
- Increasing the number of undulations decreases the rate of heat transfer. Furthermore, increasing N causes in decreasing of the convection flow and the stream function.
- The $\overline{Nu}_{hnf}$ is heavily influenced by Ra* and the number of undulations. As Ra* increases, $\overline{Nu}_{hnf}$ increases, while it decreases as the number of undulations increases.

The G-FEM could be applied to a variety of physical and technical challenges in the future^60–68^.

## Date availability

All data generated or analyzed during this study are included in this published article.

## List of symbols

*A*: The amplitude of the wavy wall
*Cp*: Specific heat (J kg^−^^1^ K^−^^1^)
Da: Darcy number
*g*: Gravitational acceleration (m s^−^^2^)
*k*: Thermal conductivity (W m^−^^1^ K^−^^1^)
*kr*: Thermal conductivity ratio
*L*: Non-dimensional Length of the enclosure
*N*: Number of undulations
Nu: Nusselt number
*P*: Non-dimensional pressure
Pr: Prandtl number
Ra: Rayleigh number
Ra*: Modified Rayleigh number
*T*: Dimensional temperature (K)
*U*: Non-dimensional velocity component X-direction
*V*: Non-dimensional velocity component Y-direction
*X*: Non-dimensional X-coordinates
*Y*: Non-dimensional Y-coordinates

## Greek symbols

*Ψ*: Absolute stream function
*ε*: Porosity
*ϕ*: Solid volume fraction
*θ*: Dimensionless temperature
*ρ*: Density (J kg^−^^1^ K^−^^1^)
*µ*: Dynamic viscosity (kg m^−^^1^ s^−^^1^)
*α*: Thermal diffusivity (m^2^ s^−^^1^)
*β*: Thermal expansion coefficient (K^−^^1^)

## Subscripts

*avg*: Average
*c*: Cold
*f*: Fluid (pure water)
*h*: Hot
*L*: Local
*hnf*: Hybrid nanofluid
